# Numerical solutions and error estimations for the space fractional diffusion equation with variable coefficients via Fibonacci collocation method

**DOI:** 10.1186/s40064-016-2853-6

**Published:** 2016-08-22

**Authors:** Ayşe Kurt Bahşı, Salih Yalçınbaş

**Affiliations:** Department of Mathematics, Manisa Celal Bayar University, Manisa, Turkey

**Keywords:** Fibonacci polynomials, Fractional diffusion equations, Collocation method, Matrix relations, Error estimation algorithm

## Abstract

In this study, the Fibonacci collocation method based on the Fibonacci polynomials are presented to solve for the fractional diffusion equations with variable coefficients. The fractional derivatives are described in the Caputo sense. This method is derived by expanding the approximate solution with Fibonacci polynomials. Using this method of the fractional derivative this equation can be reduced to a set of linear algebraic equations. Also, an error estimation algorithm which is based on the residual functions is presented for this method. The approximate solutions are improved by using this error estimation algorithm. If the exact solution of the problem is not known, the absolute error function of the problems can be approximately computed by using the Fibonacci polynomial solution. By using this error estimation function, we can find improved solutions which are more efficient than direct numerical solutions. Numerical examples, figures, tables are comparisons have been presented to show efficiency and usable of proposed method.

## Background

In recent years, many studies have been developed about fractional partial differential equations. Fractional partial differential equations arise in many physical and engineering problems such as fractional order diffusion equations which are generalizations of classical diffusion equations and modelling of linear vibrations of axial moving systems (Saadatmandi and Dehghan 2011; Dönmez Demir et al. 2013).

In this paper, we consider the one-dimensional space fractional diffusion equation with variable coefficients

1 $$\frac{{\partial u\left( {x,t} \right)}}{\partial t} = c\left( x \right)\frac{{\partial^{\alpha } u\left( {x,t} \right)}}{{\partial x^{\alpha } }} + q\left( {x,t} \right), \quad 0 < x < l,\quad 0 \le t \le \tau ,\quad 1 < \alpha \le 2,$$

with the initial condition

$$u\left( {x,0} \right) = k\left( x \right),\quad 0 < x < l,$$

and the boundary conditions

$$\begin{aligned} u\left( {0,t} \right) & = g_{0} \left( t \right), \quad 0 < t \le \tau , \\ u\left( {l,t} \right) & = g_{1} \left( t \right),\quad 0 < t \le \tau , \\ \end{aligned}$$

where *c*(*x*) ≠ 0 is diffusion coefficient, *q*(*x*, *t*), *k*(*x*), *g*_0_(*t*) and *g*_1_(*t*) are known functions and the function *u*(*x*, *t*) is unknown.

Some authors interested in the numerical solution of Eq. (1) by using various methods such as Tau method (Saadatmandi and Dehghan 2011), Chebyshev collocation method (Khader 2011), spatial extrapolation (Tadjeran et al. 2006) and finite difference method (Dehghan 2006).

## Fibonacci polynomials

In this study, we introduce Fibonacci collocation method based on matrix relations which has been used to find the approximate solutions of some classes of the differential equations such as integro-differential equations, differential-difference equations, Fredholm integro differential-difference equations, Pantograph-type functional differential equations and linear Volterra integro differential equations. Fibonacci collocation method is presented for the solution of mth-order linear differential-difference equations with variable coefficients under the mixed conditions (Kurt et al. 2013a, b) and this method is also used to solve both the linear Fredholm integro-differential-difference equations (Kurt et al. 2013a, b) and high-order Pantograph-type functional differential equations (Kurt Bahşı et al. 2015). On the other hand this method is applied for the linear Volterra integro differential equations (Kurt Bahşı and Yalçınbaş 2016a, b). Finally the mentioned method is presented for the class of the partial differential equations which are called Telegraph equations (Kurt Bahşı and Yalçınbaş 2016a, b). Also recently there are several using the other collocation methods to solve different types of the partial differential equations by using various special polynomials without Fibonacci polynomials such as Taylor (Bülbül and Sezer 2011), Bessel (Yüzbaşı and Şahin 2013), Chebyshev (Yüksel and Sezer 2014), Bernstein (Isik et al. 2014) and Bernoulli (Erdem Biçer and Yalçinbas 2016). On the other hand, these matrix methods were used for the numerical analysis of the longitudinal vibration of rods (Çevik 2010). By developing the Fibonacci collocation method, we will obtain the approximate solution of Eq. (1) in the truncated Fibonacci series form

2 $$u_{N} \left( {x,t} \right) = \mathop \sum \limits_{m = 1}^{N} \mathop \sum \limits_{n = 1}^{N} a_{mn} F_{m} \left( x \right)F_{n} \left( t \right)$$

where *a*_*mn*_; *m*, *n* = 1, …, *N* are the unknown Fibonacci coefficients and *F*_*n*_(*x*), *F*_*m*_(*x*); *m*, *n* = 1, …, *N* are the Fibonacci functions defined by,

$$F_{n} \left( x \right) = \mathop \sum \limits_{j = 0}^{{\left[ {\frac{{\left( {n - 1} \right)}}{2}} \right]}} \left( {\begin{array}{*{20}c} {n - j - 1} \\ j \\ \end{array} } \right)x^{n - 2j - 1} , \quad \left[ {\left( {n - 1} \right)/2} \right] = \left\{ {\begin{array}{*{20}c} {\frac{n - 2}{2},\quad n \,even } \\ {\frac{n - 1}{2},\quad n\, odd} \\ \end{array} } \right..$$

## The fractional derivative in the Caputo sense

### **Definition 1**

Caputo’s definition of the fractional-order derivative is defined as

$$D^{\alpha } f\left( x \right) = \frac{1}{{\varGamma \left( {n - \alpha } \right)}}\mathop \int \limits_{0}^{x} \frac{{f^{\left( n \right)} \left( t \right)}}{{\left( {x - t} \right)^{\alpha + 1 - n} }}dt, \quad n - 1 < \alpha < n,\quad n \in {\mathbb{N}}$$

where *α* > 0 is the order of derivative, Γ(.) is the Gamma function and *n* = [*α*] + 1, with [*α*] denoting the integral part of *α*.

Recall that for $\alpha \in {\mathbb{N}}$, the Caputo differential operator coincides with the usual differential operator of integer order. For the Caputo’s derivative we have (Diethelm 2010),

$$\begin{aligned} D^{\alpha } C & = 0,\quad \left( {C\, {\text{is a constant}}} \right) \\ D^{\alpha } x^{\beta } & = \left\{ {\begin{array}{*{20}l} {0,} \hfill &\quad {{\text{for}} \,\beta \in {\mathbb{N}}_{0} \,{\text{and}} \,\beta < \left[ \alpha \right].} \hfill \\ {\frac{{\varGamma \left( {\beta + 1} \right)}}{{\varGamma \left( {\beta + 1 - \alpha } \right)}},} \hfill &\quad \begin{aligned} &{\text{for}} \,\beta \in {\mathbb{N}}_{0} \,{\text{and}}\, \beta \notin {\mathbb{N}}\,{\text{and}} \hfill \\ &\beta > \left[ \alpha \right]. \hfill \\ \end{aligned} \hfill \\ \end{array} } \right. \\ \end{aligned}$$

We use the ceiling function [*α*] to denote the smallest integer greater than or equal to *α*, the floor function [*α*] to denote the largest integer less than or equal to *α* and β > [*α*] to denote order of *x*.

We use also ${\mathbb{N}} = \left\{ {1, 2, \ldots } \right\}$ and ${\mathbb{N}}_{0} = \left\{ {0, 1, 2, \ldots } \right\}$. Similar to integer-order differentiation, Caputo’s fractional differentiation is a linear operator.

## Fundamental matrix relations

In this part, we have given some fundamental matrix relations for transforming Eq. (1) to matrix equation forms.

To obtain the numerical solution of the one-dimensional space fractional diffusion problem by using Fibonacci polynomials, we first evaluate the Fibonacci coefficients of the unknown function. The approximate solution (2) can be written in the matrix form

3 $$u_{N} \left( {x,t} \right) = {\mathbf{F}}\left( x \right)\overline{{\mathbf{F}}} \left( t \right){\mathbf{A}}$$

where

$$\begin{aligned} {\mathbf{F}}\left( x \right) & = \left[ {\begin{array}{*{20}c} {F_{1} \left( x \right)} & {F_{2} \left( x \right)} & \cdots & {F_{N} \left( x \right)} \\ \end{array} } \right]_{1 \times N} , \\ \overline{{\mathbf{F}}} \left( t \right) & = \left[ {\begin{array}{*{20}c} {{\mathbf{F}}\left( t \right)} & {\mathbf{0}} & \cdots & {\mathbf{0}} \\ {\mathbf{0}} & {{\mathbf{F}}\left( t \right)} & \cdots & {\mathbf{0}} \\ \vdots & \vdots & \ddots & \vdots \\ {\mathbf{0}} & {\mathbf{0}} & \cdots & {{\mathbf{F}}\left( t \right)} \\ \end{array} } \right]_{{N \times N^{2} }} , \\ \end{aligned}$$

and

$${\mathbf{A}} = \left[ {\begin{array}{*{20}c} {{\mathbf{A}}_{1} } & {{\mathbf{A}}_{2} } & \cdots & {{\mathbf{A}}_{N} } \\ \end{array} } \right]_{{1 \times N^{2} }}^{T}$$

such that

$${\mathbf{A}}_{i} = \left[ {\begin{array}{*{20}c} {a_{i1} } & {a_{i2} } & \cdots & {a_{iN} } \\ \end{array} } \right]_{1 \times N}^{T} , \quad i = 1, 2, \ldots ,N.$$

In here, the matrix form **F**(*x*) can be written as

4 $${\mathbf{F}}\left( x \right) = {\mathbf{X}}\left( x \right){\mathbf{C}}^{T}$$

so that

$${\mathbf{X}}\left( x \right) = \left[ {\begin{array}{*{20}c} 1 & x & \cdots & {x^{N - 1} } \\ \end{array} } \right].$$

If *N* is even,

$${\mathbf{C}} = \left[ {\begin{array}{*{20}c} {\left( {\begin{array}{*{20}c} 0 \\ 0 \\ \end{array} } \right)} & 0 & 0 & 0 & \cdots & 0 \\ 0 & {\left( {\begin{array}{*{20}c} 1 \\ 0 \\ \end{array} } \right)} & 0 & 0 & \cdots & 0 \\ {\left( {\begin{array}{*{20}c} 1 \\ 1 \\ \end{array} } \right)} & 0 & {\left( {\begin{array}{*{20}c} 2 \\ 0 \\ \end{array} } \right)} & 0 & \cdots & 0 \\ 0 & {\left( {\begin{array}{*{20}c} 2 \\ 1 \\ \end{array} } \right)} & 0 & {\left( {\begin{array}{*{20}c} 3 \\ 0 \\ \end{array} } \right)} & \cdots & 0 \\ \vdots & \vdots & \vdots & \vdots & \vdots & \vdots \\ {\left( {\begin{array}{*{20}c} {\left( {n - 2} \right)/2} \\ {\left( {n - 2} \right)/2} \\ \end{array} } \right)} & 0 & {\left( {\begin{array}{*{20}c} {n/2} \\ {\left( {n - 4} \right)/2} \\ \end{array} } \right)} & 0 & \cdots & 0 \\ 0 & {\left( {\begin{array}{*{20}c} {n/2} \\ {\left( {n - 2} \right)/2} \\ \end{array} } \right)} & 0 & {\left( {\begin{array}{*{20}c} {\left( {n + 2} \right)/2} \\ {\left( {n - 4} \right)/2} \\ \end{array} } \right)} & \cdots & {\left( {\begin{array}{*{20}c} {n - 1} \\ 0 \\ \end{array} } \right)} \\ \end{array} } \right].$$

If *N* is odd,

$${\mathbf{C}} = \left[ {\begin{array}{*{20}c} {\left( {\begin{array}{*{20}c} 0 \\ 0 \\ \end{array} } \right)} & 0 & 0 & 0 & \cdots & 0 \\ 0 & {\left( {\begin{array}{*{20}c} 1 \\ 0 \\ \end{array} } \right)} & 0 & 0 & \cdots & 0 \\ {\left( {\begin{array}{*{20}c} 1 \\ 1 \\ \end{array} } \right)} & 0 & {\left( {\begin{array}{*{20}c} 2 \\ 0 \\ \end{array} } \right)} & 0 & \cdots & 0 \\ 0 & {\left( {\begin{array}{*{20}c} 2 \\ 1 \\ \end{array} } \right)} & 0 & {\left( {\begin{array}{*{20}c} 3 \\ 0 \\ \end{array} } \right)} & \cdots & 0 \\ \vdots & \vdots & \vdots & \vdots & \vdots & \vdots \\ 0 & {\left( {\begin{array}{*{20}c} {\left( {n - 1} \right)/2} \\ {\left( {n - 3} \right)/2} \\ \end{array} } \right)} & 0 & {\left( {\begin{array}{*{20}c} {\left( {n + 1} \right)/2} \\ {\left( {n - 5} \right)/2} \\ \end{array} } \right)} & \cdots & 0 \\ {\left( {\begin{array}{*{20}c} {\left( {n - 1} \right)/2} \\ {\left( {n - 1} \right)/2} \\ \end{array} } \right)} & 0 & {\left( {\begin{array}{*{20}c} {\left( {n + 1} \right)/2} \\ {\left( {n - 3} \right)/2} \\ \end{array} } \right)} & 0 & \cdots & {\left( {\begin{array}{*{20}c} {n - 1} \\ 0 \\ \end{array} } \right)} \\ \end{array} } \right]\varvec{ }$$

**C** is the characteristic matrix of the matrix relations (Kurt et al. 2013a, b). On the other hand, we can express the relations

5 $$\overline{{\mathbf{F}}} \left( t \right) = \overline{{\mathbf{X}}} \left( t \right)\overline{{\mathbf{C}}}^{T}$$

where

$$\overline{{\mathbf{X}}} \left( t \right) = \left[ {\begin{array}{*{20}c} {{\mathbf{X}}\left( t \right)} \\ {\mathbf{0}} \\ {\begin{array}{*{20}c} \vdots \\ {\mathbf{0}} \\ \end{array} } \\ \end{array} \varvec{ }\begin{array}{*{20}c} {\mathbf{0}} & \cdots & {\mathbf{0}} \\ {{\mathbf{X}}\left( t \right)} & \cdots & {\mathbf{0}} \\ {\begin{array}{*{20}c} \vdots \\ \cdots \\ \end{array} } & {\begin{array}{*{20}c} \ddots \\ {\mathbf{0}} \\ \end{array} } & {\begin{array}{*{20}c} \vdots \\ {{\mathbf{X}}\left( t \right)} \\ \end{array} } \\ \end{array} } \right]$$

and

$$\varvec{ }\overline{{\mathbf{C}}}^{T} = \left[ {\begin{array}{*{20}c} {\varvec{ }{\mathbf{C}}^{\varvec{T}} } \\ {\mathbf{0}} \\ {\begin{array}{*{20}c} \vdots \\ {\mathbf{0}} \\ \end{array} } \\ \end{array} \varvec{ }\begin{array}{*{20}c} {\mathbf{0}} & \cdots & {\mathbf{0}} \\ {\varvec{ }{\mathbf{C}}^{\varvec{T}} } & \cdots & {\mathbf{0}} \\ {\begin{array}{*{20}c} \vdots \\ {\mathbf{0}} \\ \end{array} } & {\begin{array}{*{20}c} \ddots \\ \cdots \\ \end{array} } & {\begin{array}{*{20}c} \vdots \\ {\varvec{ }{\mathbf{C}}^{\varvec{T}} } \\ \end{array} } \\ \end{array} } \right].$$

Firstly from the relations (3–5), we can obtain the desired solution *u*(*x*, *t*) of Eq. (1) defined by the truncated Fibonacci series (2) in matrix form as follow

6 $$u\left( {x,t} \right) = {\mathbf{X}}\left( x \right){\mathbf{C}}^{T} \overline{{\mathbf{X}}} \left( t \right) \overline{{\mathbf{C}}}^{T}{\mathbf{A}}.$$

And secondly we can define the matrix form of the partial derivatives for each independent variables of *u*(*x*, *t*) term can be written as

7 $$u_{x} \left( {x,t} \right) = {\mathbf{X}}\left( x \right) {\mathbf{B}}\varvec{ }{\mathbf{C}}^{T} \overline{{\mathbf{X}}} \left( t \right)\overline{{\mathbf{C}}}^{T} {\mathbf{A}}$$

8 $$u_{t} \left( {x,t} \right) = {\mathbf{X}}\left( x \right)\varvec{ }{\mathbf{C}}^{T} \overline{{\mathbf{X}}} \left( t \right)\overline{\varvec{B}}\,\, \overline{{\mathbf{C}}}^{T} {\mathbf{A}}$$

where

$${\mathbf{B}} = \left[ {\begin{array}{*{20}c} {\begin{array}{*{20}c} 0 &\quad 1 &\quad 0 \\ 0 &\quad 0 &\quad 2 \\ \end{array} } &\quad \cdots &\quad {\begin{array}{*{20}c} 0 \\ 0 \\ \end{array} } \\ \vdots &\quad \ddots &\quad \vdots \\ {\begin{array}{*{20}c} 0 &\quad 0 &\quad 0 \\ 0 &\quad 0 &\quad 0 \\ \end{array} } &\quad \cdots &\quad {\begin{array}{*{20}c} {N - 1} \\ 0 \\ \end{array} } \\ \end{array} } \right]$$

and

$$\overline{{\mathbf{B}}} = \left[ {\begin{array}{*{20}c} {\mathbf{B}} \\ {\mathbf{0}} \\ {\begin{array}{*{20}c} \vdots \\ {\mathbf{0}} \\ \end{array} } \\ \end{array} \begin{array}{*{20}c} {\mathbf{0}} & \cdots & {\mathbf{0}} \\ {\mathbf{B}} & \cdots & {\mathbf{0}} \\ {\begin{array}{*{20}c} \vdots \\ {\mathbf{0}} \\ \end{array} } & {\begin{array}{*{20}c} \ddots \\ \cdots \\ \end{array} } & {\begin{array}{*{20}c} \vdots \\ {\mathbf{B}} \\ \end{array} } \\ \end{array} } \right].$$

We introduce the relation between the matrix **X**(*x*) and its derivatives **X**′(*x*) which can be expressed as

$${\mathbf{X}}^{\prime } \left( x \right) = {\mathbf{X}}\left( x \right){\mathbf{B}}.$$

Similarly, the relation between the matrix **X**(*t*)and its derivative **X′**(*t*) is written

$$\overline{{\mathbf{X}}}^{\prime } \left( t \right) = \varvec{ }\overline{{\mathbf{X}}} \left( t \right)\overline{{\mathbf{B}}} \varvec{ }.$$

Finally, we can explain the matrix form of $\frac{{\partial^{\alpha } u\left( {x,t} \right)}}{{\partial x^{\alpha } }}$ which is the fractional derivative of *u*(*x*, *t*) term, can be written as

9 $$\left[ {\frac{{\partial^{\alpha } u\left( {x,t} \right)}}{{\partial x^{\alpha } }} } \right] = \varvec{ }{\mathbf{M}}\left( x \right)\varvec{ }{\mathbf{C}}^{\varvec{T}} \overline{{\mathbf{X}}} \left( t \right)\varvec{ }\overline{{\mathbf{C}}}^{T} {\mathbf{A}}$$

where

$${\mathbf{M}}\left( x \right) = \frac{{\partial^{\alpha } {\mathbf{X}}\left( x \right)}}{{\partial x^{\alpha } }} = \left[ {\begin{array}{*{20}c} 0 & {D^{\alpha } x} & {D^{\alpha } x^{2} } & \cdots & {D^{\alpha } x^{N - 1} } \\ \end{array} } \right]_{1 \times N}$$

such that

$$D^{\alpha } x^{i} = \frac{{\varGamma \left( {i + 1} \right)}}{{\varGamma \left( {i + 1 - \alpha } \right)}}x^{i - \alpha } , \quad i = 1, \ldots , N - 1.$$

By using the relations (7), (8), (9) into Eq. (1), we can have the closed matrix form

$$\underbrace {{{\mathbf{X}}\left( x \right)\varvec{ }{\mathbf{C}}^{T} \overline{{\mathbf{X}}} \left( t \right) \overline{{\mathbf{B}}} \,\,\overline{{\mathbf{C}}}^{T} - c\left( x \right)\varvec{ }{\mathbf{M}}\left( x \right) {\mathbf{C}}^{\varvec{T}} \varvec{ }\overline{{\mathbf{X}}} \left( t \right)\overline{{\mathbf{C}}}^{T} {\mathbf{A}}}}_{{{\mathbf{W}}\left( {x,t} \right)}} = q\left( {x,t} \right).$$

Briefly form is written

10 $${\mathbf{W}}\left( {x,t} \right){\mathbf{A}} = q\left( {x,t} \right).$$

We have the corresponding matrix forms for the initial condition and for the boundary conditions (1) by means of the relation (6)

$$\begin{aligned} u\left( {x,0} \right) = {\mathbf{X}}\left( x \right)\varvec{ }{\mathbf{C}}^{\varvec{T}} \overline{{\mathbf{X}}} \left( 0 \right)\overline{{\mathbf{C}}}^{T} {\mathbf{A}} = k\left( x \right) \hfill \\ u\left( {0,t} \right) = {\mathbf{X}}\left( 0 \right) {\mathbf{C}}^{T} \overline{{\mathbf{X}}} \left( t \right)\overline{{\mathbf{C}}}^{T} {\mathbf{A}} = g_{0} \left( t \right) \hfill \\ u\left( {l,t} \right) = {\mathbf{X}}\left( l \right) {\mathbf{C}}^{T} \overline{{\mathbf{X}}} \left( t \right) \overline{{\mathbf{C}}}^{T} {\mathbf{A}} = g_{1} \left( t \right) \hfill \\ \end{aligned}$$

## Collocation method

For the matrix equation Eq. (10) by using collocation points defined by

$$\begin{aligned} x_{i} & = \frac{l}{N - 1}\left( {i - 1} \right),\quad i = 1, 2, \ldots , N \\ t_{j} & = \frac{\tau }{N - 1}\left( {j - 1} \right),\quad j = 1, 2, \ldots , N. \\ \end{aligned}$$

We obtain the system of matrix equations

$${\mathbf{W}}\left( {x_{i} ,t_{j} } \right){\mathbf{A}} = q\left( {x_{i} ,t_{j} } \right),\quad i = 1, 2, \ldots , N,\quad j = 1, 2, \ldots , N.$$

Using the system of matrix equation, the fundamental matrix equation becomes

$${\mathbf{WA}} = {\mathbf{Q}}\quad {\text{or}}\quad \left[ {{\mathbf{W}};{\mathbf{Q}}} \right]$$

The fundamental matrix Eq. (4) of Eq. (1) corresponds to a system of *N*^2^ algebraic equations for the *N*^2^ unknown coefficients *a*_*mn*_; *m*, *n* = 1, 2, …, *N*. On the other hand, by using collocation points, we can obtain matrix form of the initial condition

$$\begin{aligned} & {\mathbf{U}}_{1} {\mathbf{A}} = {\mathbf{K}} \\ & {\mathbf{U}}_{1} = \left[ {{\mathbf{X}}\left( x \right)\varvec{ }{\mathbf{C}}^{\varvec{T}} \varvec{ }\overline{{\mathbf{X}}} \left( 0 \right)\overline{{\mathbf{C}}}^{\varvec{T}} } \right],\quad {\mathbf{K}} = \left[ {k\left( {x_{i} } \right)} \right]_{N \times 1} ,\quad i = 1, 2, \ldots , N \\ \end{aligned}$$

and matrix form of the boundary conditions as follow

$$\begin{aligned} & {\mathbf{U}}_{2} {\mathbf{A}} = {\mathbf{G}}_{0} \\ & {\mathbf{U}}_{2} = \left[ {{\mathbf{X}}\left( 0 \right)\varvec{ }{\mathbf{C}}^{\varvec{T}} \overline{{\mathbf{X}}} \left( t \right)\varvec{ }\overline{{\mathbf{C}}} } \right],\quad {\mathbf{G}}_{0} = \left[ {g_{0} \left( {t_{j} } \right)} \right]_{N \times 1} ,\quad j = 1, 2, \ldots , N \\ \end{aligned}$$

and

$$\begin{aligned} & {\mathbf{U}}_{3} {\mathbf{A}} = {\mathbf{G}}_{1} \\ & {\mathbf{U}}_{3} = \left[ {{\mathbf{X}}\left( l \right)\varvec{ }{\mathbf{C}}^{\varvec{T}} \overline{{\mathbf{X}}} \left( t \right)\overline{{\mathbf{C}}}^{\varvec{T}} } \right],\quad {\mathbf{G}}_{1} = \left[ {g_{1} \left( {t_{j} } \right)} \right]_{N \times 1} ,\quad j = 1, 2, \ldots , N. \\ \end{aligned}$$

Subsequently, in order to obtain the solution of Eq. (1) under the initial and boundary conditions we can write augmented matrix which is contain all components of this problem

$$\left[ {{\tilde{\mathbf{W}}};{\tilde{\mathbf{Q}}}} \right] = \left[ {\begin{array}{*{20}c} {{\mathbf{U}}_{1} } & ; & {\mathbf{K}} \\ {{\mathbf{U}}_{2} } & ; & {{\mathbf{G}}_{0} } \\ {{\mathbf{U}}_{3} } & ; & {{\mathbf{G}}_{1} } \\ {\mathbf{W}} & ; & {\mathbf{Q}} \\ \end{array} } \right].$$

So, the unknown Fibonacci coefficients are obtained as

$${\mathbf{A}} = \left( {{\tilde{\tilde{\mathbf{W}}}}} \right)^{ - 1} {\tilde{\tilde{\mathbf{Q}}}}$$

where $$\left[ {\begin{array}{*{20}c} {{\tilde{\tilde{\mathbf{W}}}}} & ; & {{\tilde{\tilde{\mathbf{Q}}}}} \\ \end{array} } \right]$$ is generated by using the Gauss elimination method and then removing zero rows of the augmented matrix $$\left[ {\begin{array}{*{20}c} {{\tilde{\mathbf{W}}}} & ; & {{\tilde{\mathbf{Q}}}} \\ \end{array} } \right]$$.

## Error estimation algorithm and analyses

In this section, we will give an efficient error estimation for the Fibonacci polynomial approximation and also a technique to obtain the corrected solution of the problem (1) by using the residual correction method (Oliveira 1980; Shahmorad 2005; Çelik 2006). For our aim, we define the residual function for the present method as

$$R_{N} \left( {x,t} \right) = L\left[ {u_{N} \left( {x,t} \right)} \right] - q\left( {x,t} \right)$$

where

$$L\left[ {u_{N} \left( {x,t} \right)} \right] = \frac{{\partial u_{N} \left( {x,t} \right)}}{\partial x} - c\left( x \right)\frac{{\partial^{\alpha } u_{N} \left( {x,t} \right)}}{{\partial x^{\alpha } }} .$$

Note that, Fibonacci polynomial solution satisfies the following problem

11 $$L\left[ {u_{N} \left( {x,t} \right)} \right] = \frac{{\partial u_{N} \left( {x,t} \right)}}{\partial x} - c\left( x \right)\frac{{\partial^{\alpha } u_{N} \left( {x,t} \right)}}{{\partial x^{\alpha } }} = q\left( {x,t} \right) + R_{N} \left( {x,t} \right)$$

with the initial and boundary conditions

12 $$\begin{aligned} u_{N} \left( {x,0} \right) & = k\left( x \right), \quad 0 < x < l \\ u_{N} \left( {0,t} \right) & = g_{0} \left( t \right),\quad 0 < t \le \tau \\ u_{N} \left( {l,t} \right) & = g_{1} \left( t \right), \quad 0 < t \le \tau . \\ \end{aligned}$$

Furthermore, the error function *e*_*N*_(*x*, *t*) can be defined as

13 $$e_{N} \left( {x,t} \right) = u\left( {x,t} \right) - u_{N} \left( {x,t} \right)$$

where *u*(*x*, *t*) is the exact solution of the problem (1).

By using Eqs. (1) and (11–13), we have the error differential equation

$$L\left[ {e_{N} \left( {x,t} \right)} \right] = L\left[ {u\left( {x,t} \right)} \right] - L\left[ {u_{N} \left( {x,t} \right)} \right] = - R_{N} \left( {x,t} \right)$$

with the homogenous conditions

$$\begin{aligned} e_{N} \left( {x,0} \right) & = 0, \quad 0 < x < l \\ e_{N} \left( {0,t} \right) & = 0,\quad 0 < t \le \tau \\ e_{N} \left( {l,t} \right) & = 0, \quad 0 < t \le \tau . \\ \end{aligned}$$

Subsequently the error problem can be written as

14 $$\begin{aligned} & \frac{{\partial e_{N} \left( {x,t} \right)}}{\partial x} - c\left( x \right)\frac{{\partial^{\alpha } e_{N} \left( {x,t} \right)}}{{\partial x^{\alpha } }} = - R_{N} \left( {x,t} \right) \\ & e_{N} \left( {x,0} \right) = 0,\quad 0 < x < l \\ & e_{N} \left( {0,t} \right) = 0,\quad 0 < t \le \tau \\ & e_{N} \left( {l,t} \right) = 0,\quad 0 < t \le \tau . \\ \end{aligned}$$

Solving the problem (14) in the same way as in “The fractional derivative in the Caputo sense” section, we get the approximation *e*_*N,M*_(*x*, *t*) to *e*_*N*_(*x*, *t*), *M* > *N* which is the error function based on the residual function. We note that if the exact solution of the problem (1) is unknown, then the error function can be estimated by *e*_*N,M*_(*x*, *t*) which is found without the exact solution and also clearly seen from given error estimation algorithm. By means of the Fibonacci polynomial solution *u*_*N*_(*x*, *t*) and error estimation function *e*_*N,M*_(*x*, *t*), we obtain the corrected Fibonacci polynomial solution

$$u_{N,M} \left( {x,t} \right) = u_{N} \left( {x,t} \right) + e_{N,M} \left( {x,t} \right).$$

## Numerical examples

In this section, two examples are given to illustrate the applicability of the Fibonacci matrix method and all of them are performed on the computer by using MAPLE symbolic program.

### *Example 1*

Consider the one-dimensional space fractional diffusion equation (Dehghan 2006)

15 $$\frac{{\partial u\left( {x,t} \right)}}{\partial t} = c\left( x \right)\frac{{\partial^{1.8} u\left( {x,t} \right)}}{{\partial x^{1.8} }} + q\left( {x,t} \right),\quad 0 < x < 1,\quad 0 < t \le 2$$

where *q*(*x*, *t*) = (6*x*^3^ − 3*x*^2^)*e*^−*t*^, diffusion coefficient *c*(*x*) = Γ(1.2)*x*^1.8^ with the initial condition

$$u\left( {x,0} \right) = x^{2} - x^{3} ,\quad 0 < x < 1$$

and the boundary conditions are

$$\begin{aligned} u\left( {0,t} \right) = 0, \hfill \\ u\left( {1,t} \right) = 0. \hfill \\ \end{aligned}$$

The exact solution of this problem *u*(*x*, *t*) = (*x*^2^ − *x*^3^)*e*^−*t*^.

**Fig. 1:**
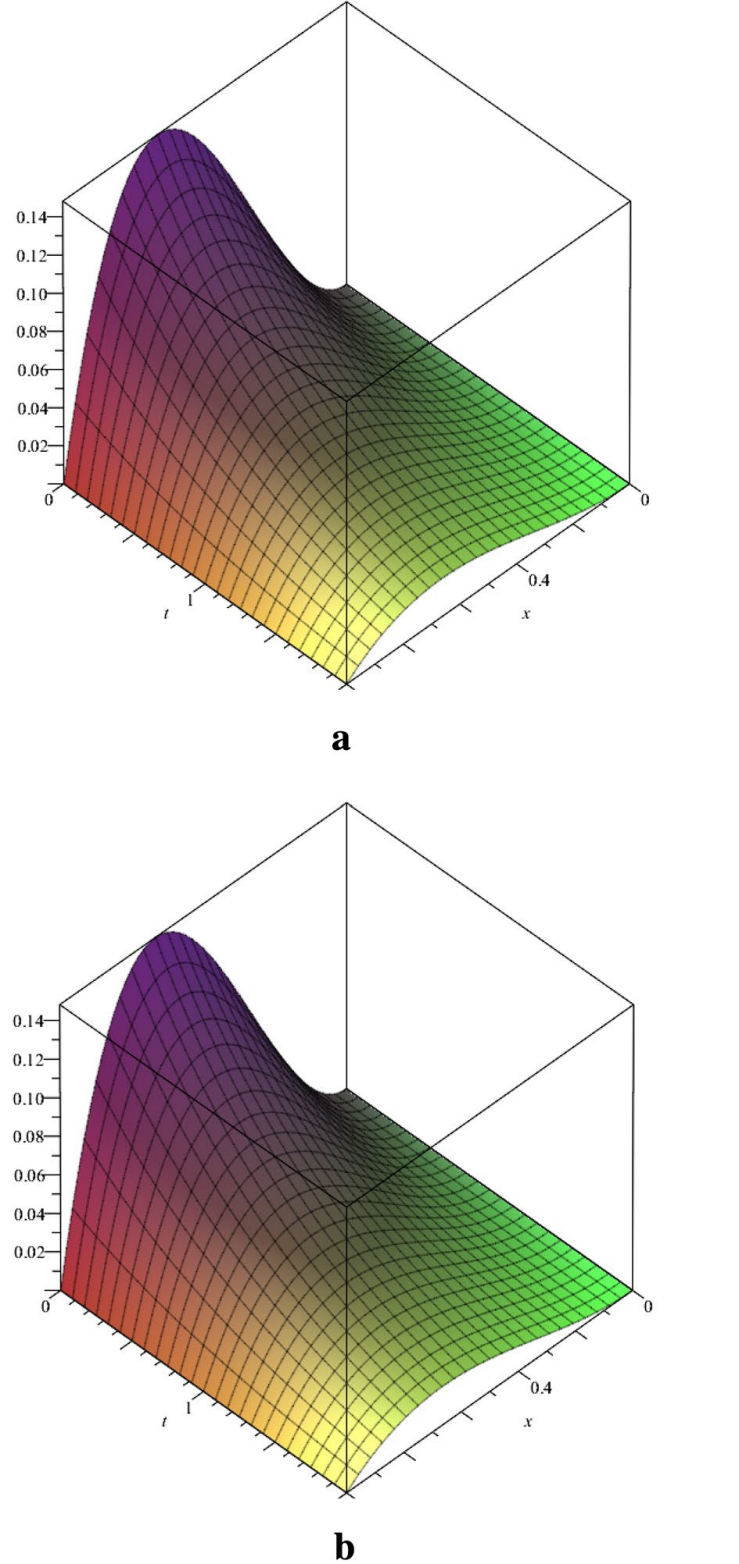
Comparison of the exact solution (**a**) and Fibonacci polynomial solution for *N* = 5 (**b**) of the Example 1

Figure 1 shows the Fibonacci polynomial solution of this problem obtained by present method for *N* = 5 with the exact solution.

In Table 1, we have presented comparison of the absolute errors of the Fibonacci polynomial solution and improved Fibonacci polynomial solution that is found by using error estimation algorithm with finite difference method (Dehghan 2006) and Tau method (Saadatmandi and Dehghan 2011) for the same order polynomial solutions at *t* = 2. We can clearly see both Fibonacci polynomial solution and improve Fibonacci polynomial solution are better approximate than the other solution for the same order polynomial solution.

**Table 1:** Comparison of the absolute error of the Fibonacci polynomial solution (*u*_*N*_(*x*, *t*)) and improved Fibonacci polynomial solution (*u*_*N,M*_(*x*, *t*)) with other methods for the same order polynomial solutions at *t* = 2 for Example 1

*x*	Finite difference method (Dehghan 2006)	Tau method (Saadatmandi and Dehghan 2011)	Present method	
	*m* = 5		*N* = 6	*N*, *M* = 6, 7
0.1	4.20*e*−5	4.47*e*−6	1.35*e*−6	1.89*e*−7
0.2	3.76*e*−5	2.78*e*−7	1.46*e*−6	4.01*e*−7
0.3	8.44*e*−5	5.81*e*−6	4.09*e*−7	5.41*e*−7
0.4	3.27*e*−5	1.02*e*−5	1.49*e*−6	6.02*e*−7
0.5	3.61*e*−5	1.17*e*−5	3.76*e*−6	6.11*e*−7
0.6	1.94*e*−5	1.08*e*−5	5.84*e*−6	5.92*e*−7
0.7	2.95*e*−5	8.54*e*−6	7.07*e*−6	5.49*e*−7
0.8	4.92*e*−5	6.06*e*−6	6.86*e*−6	4.58*e*−7
0.9	2.83*e*−5	3.67*e*−6	4.65*e*−6	2.84*e*−7

**Fig. 2:**
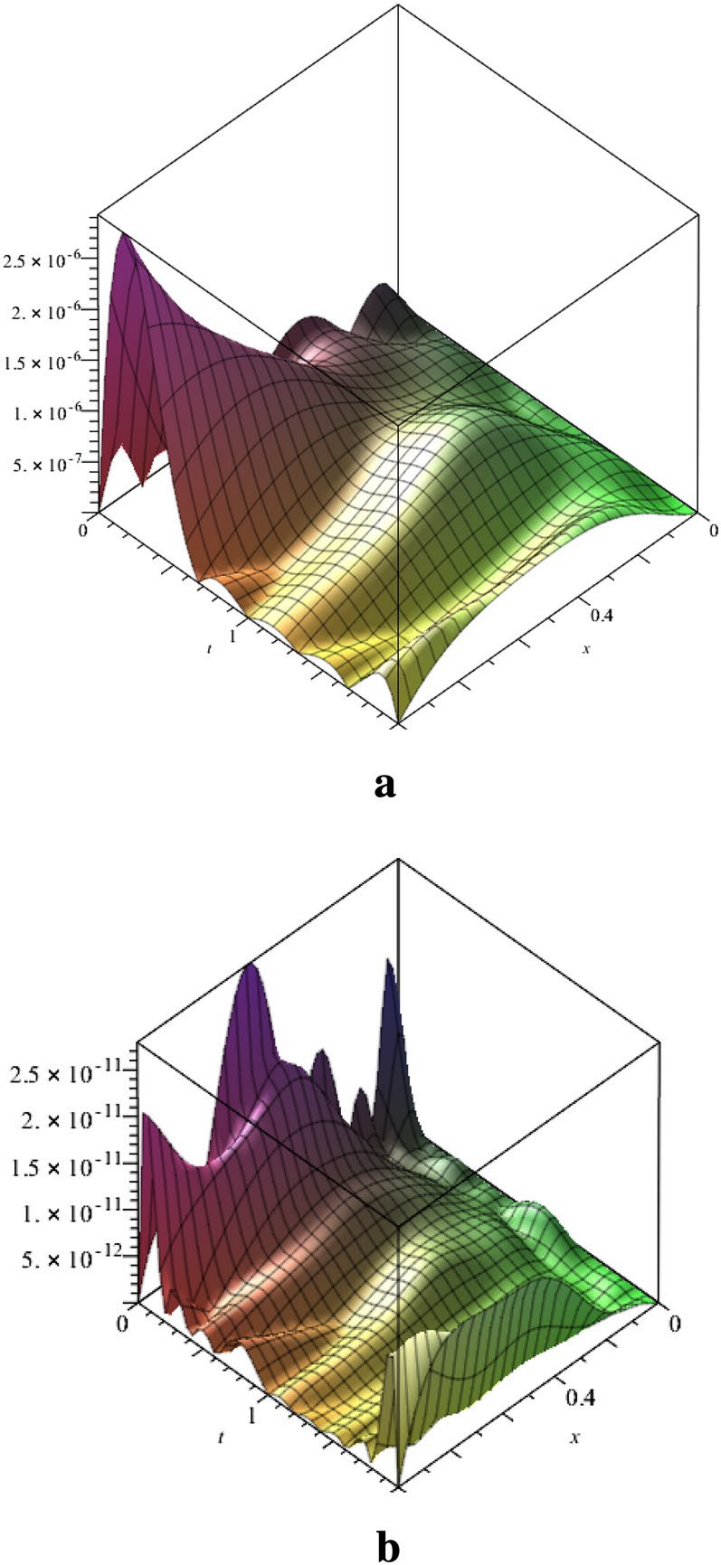
Comparison the absolute error functions of Fibonacci polynomial solution for *N* = 7 (**a**) and *N* = 11 (**b**) for the Example 1

Figure 2 is indicated the absolute error function obtained by the our method for *N* = 7, 11 and is clearly showed when *N* is increased the absolute error values is decreased in the determined domain of the problem.

### *Example* 2

In this example is the one-dimensional space fractional diffusion equation (Saadatmandi and Dehghan 2011)

16 $$\frac{{\partial u\left( {x,t} \right)}}{\partial x} = c\left( x \right)\frac{{\partial^{1.5} u\left( {x,t} \right)}}{{\partial x^{1.5} }} + q\left( {x,t} \right), \quad 0 < x < 1,\quad 0 < t \le 1$$

where *q*(*x*, *t*) = (*x*^2^ + 1)cos(*t* + 1) − 2*x*sin(*t* + 1), diffusion coefficient *c*(*x*) = Γ(1.5)*x*^0.5^ with the initial condition

$$u\left( {x,0} \right) = \left( {x^{2} + 1} \right){ \sin }\left( 1 \right), \quad 0 < x < 1$$

and the boundary conditions are

$$\begin{aligned} u\left( {0,t} \right) & = \sin \left( {t + 1} \right), \\ u\left( {1,t} \right) & = 2\sin \left( {t + 1} \right). \\ \end{aligned}$$

The exact solution of this problem *u*(*x*, *t*) = (*x*^2^ + 1) sin(*t* + 1).

**Fig. 3:**
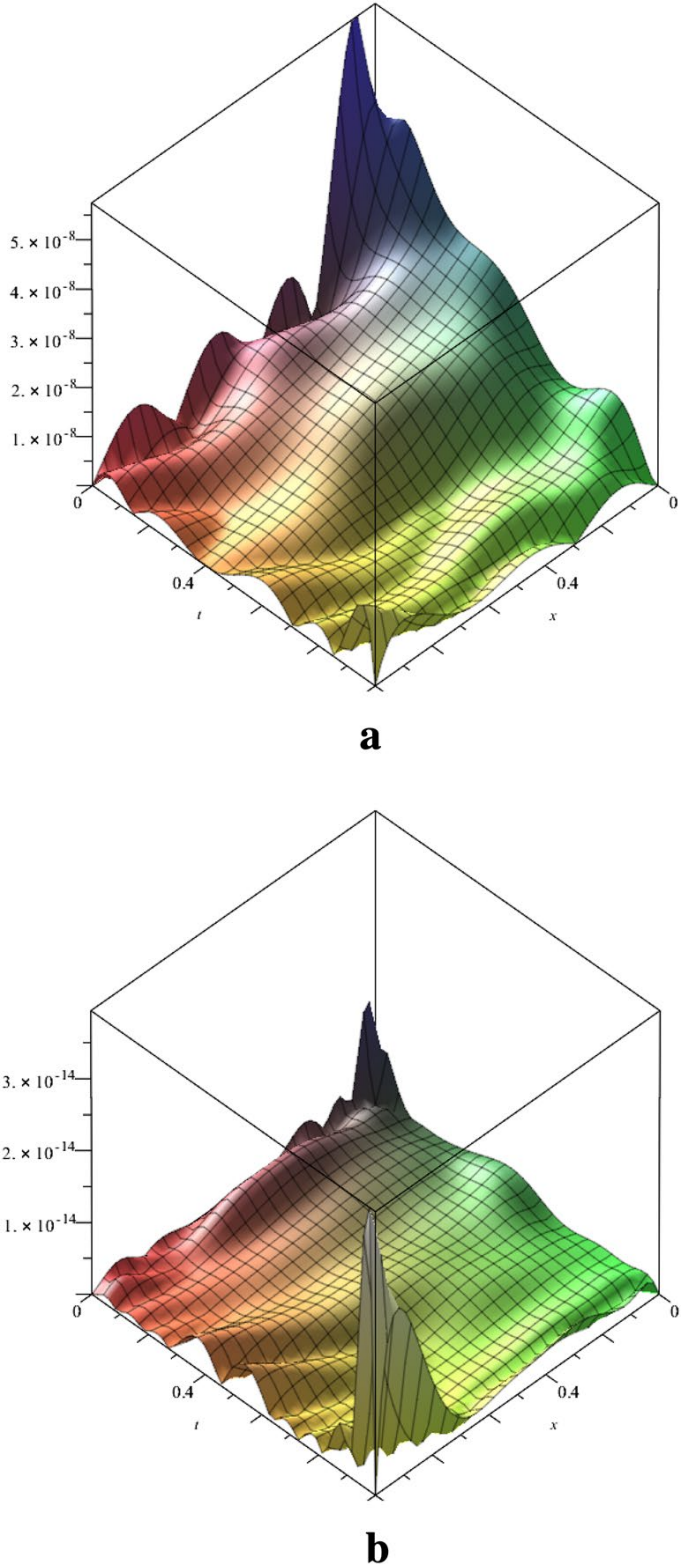
Comparison the absolute error functions of Fibonacci polynomial solution for *N* = 8 (**a**) and *N* = 12 (**b**) for the Example 2

Figure 3 shows the absolute error functions |*u*(*x*, *t*) − *u*_N_(*x*, *t*)| for the Fibonacci polynomial solutions for *N* = 8, 12. The absolute error values are decreased as *N* is increased.

The absolute errors values have given the Fibonacci collocation method are compared with Tau method (Saadatmandi and Dehghan 2011) for *t* = 1 and various values of first independent variable from 0.1 to 0.9 by 0.1 in Table 2. And also each column has given the same truncated numbers for the Tau and present methods.

**Table 2:** Comparison of the absolute error of the *u*_*N*_(*x*, *t*) with other methods for *t* = 1 for the Example 2

*x*	Tau method (Saadatmandi and Dehghan 2011)	Present method	Tau method (Saadatmandi and Dehghan 2011)	Present method
	*m* = 6		*m* = 7	
0.1	5.35*e*−5	4.19*e*−6	4.66*e*−5	9.81*e*−7
0.2	1.11*e*−4	1.11*e*−6	7.74*e*−5	1.60*e*−6
0.3	1.19*e*−4	1.89*e*−6	5.00*e*−5	1.64*e*−6
0.4	7.65*e*−5	4.40*e*−6	2.30*e*−5	1.69*e*−6
0.5	4.06*e*−5	6.04*e*−6	2.74*e*−5	1.58*e*−6
0.6	3.30*e*−5	6.74*e*−6	4.38*e*−5	1.34*e*−6
0.7	4.42*e*−5	6.46*e*−6	3.87*e*−5	1.11*e*−6
0.8	5.38*e*−5	5.20*e*−6	1.01*e*−5	9.92*e*−7
0.9	2.79*e*−5	2.99*e*−6	3.35*e*−6	8.73*e*−7

**Fig. 4:**
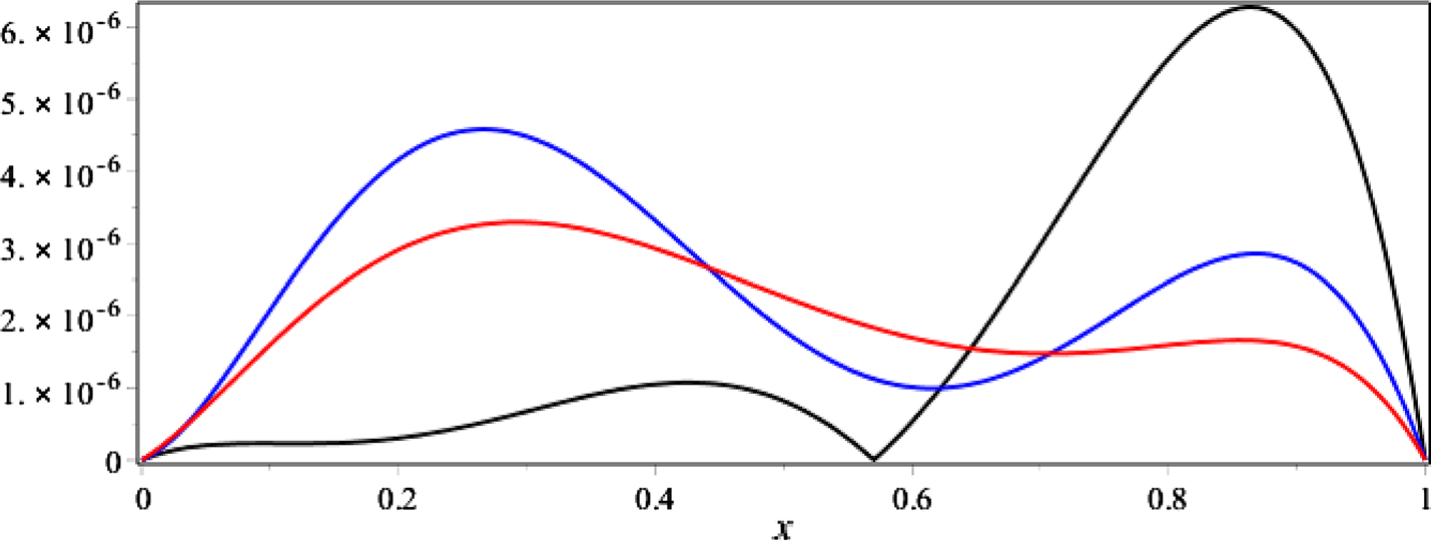
The comparison of the absolute error function |*e*_6_(*x*, 1)| (*black*) and error estimation functions |*e*_6,7_(*x*, 1)| (*blue*) and |*e*_6,8_(*x*, 1)| (*red*) for the Example 2

**Fig. 5:**
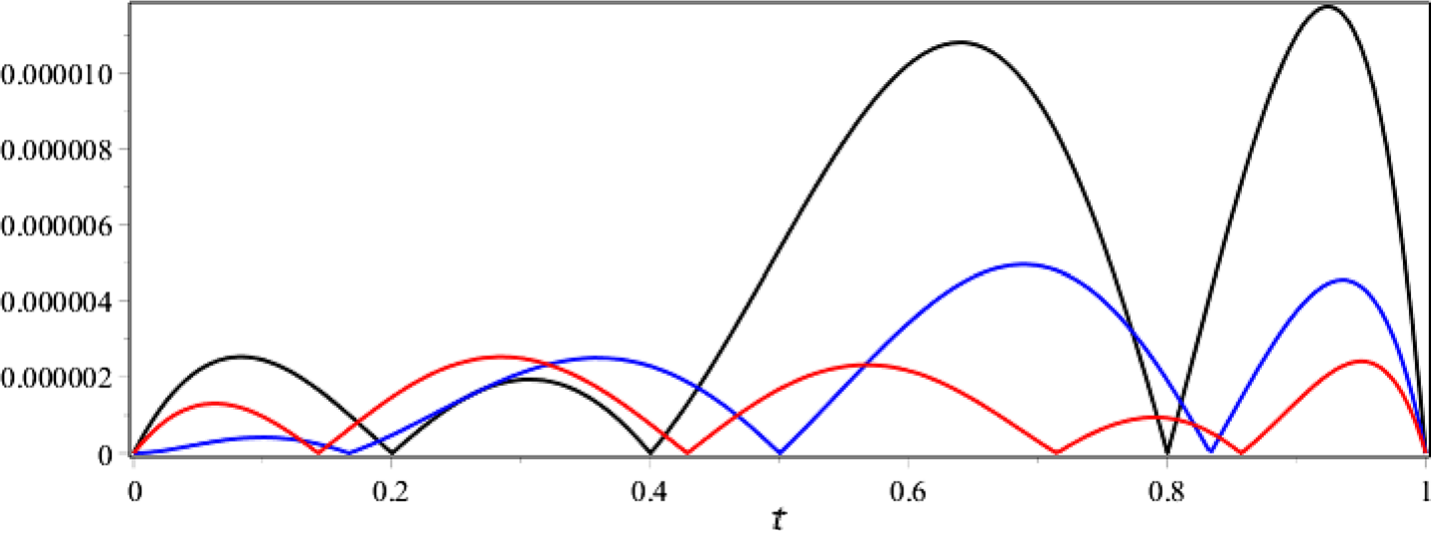
The comparison of the absolute error function |*e*_6_(1, *t*)| (*black*) and error estimation functions |*e*_6,7_(1, *t*)| (*blue*) and |*e*_6,8_(1, *t*)| (*red*) for the Example 2

Figures 4 and 5 have been compared the absolute error function of |*e*_6_(*x*, *t*)| and error estimation functions |*e*_6,7_(*x*, *t*)| and |*e*_6,8_(*x*, *t*)| with respectively *t* = 1 and *x* = 1.

## Conclusion

In this study, we have presented a new method using the Fibonacci polynomials to solve the one-dimensional space fractional diffusion equation. To this aim, we transformed the Fibonacci polynomials from algebraic form to matrix form. This method has been applied to two numerical examples which are indicated to illustrate the accuracy and efficiency. It can be observed from the results that the Fibonacci collocation method yields better approximation than the mentioned methods for the exact solution of the illustrated problems. It is observed from discussed examples which have the exact solution, the error estimation algorithm is very effective when the compared absolute errors of these examples. One of the considerable advantage of this method is that the approximate solutions are found very easily by using the symbolic programs.
